# Targeting market segment needs with public-good crop breeding investments: A case study with potato and sweetpotato focused on poverty alleviation, nutrition and gender

**DOI:** 10.3389/fpls.2023.1105079

**Published:** 2023-03-15

**Authors:** Sylvester Okoth Ojwang, Julius Juma Okello, David Jakinda Otieno, Janet Mwende Mutiso, Hannele Lindqvist-Kreuze, Peter Coaldrake, Thiago Mendes, Maria Andrade, Neeraj Sharma, Wolfgang Gruneberg, Godwill Makunde, Reuben Ssali, Benard Yada, Sarah Mayanja, Vivian Polar, Bonny Oloka, Doreen M. Chelangat, Jacqueline Ashby, Guy Hareau, Hugo Campos

**Affiliations:** ^1^ International Potato Center, Nairobi, Kenya; ^2^ International Potato Center, Kampala, Uganda; ^3^ Department of Agricultural Economics, University of Nairobi, Nairobi, Kenya; ^4^ School of International Development, University of East Anglia, Norwich, United Kingdom; ^5^ International Potato Center, Lima, Peru; ^6^ Excellence in Breeding Platform, Consultative Group on International Agricultural Research, Texcoco, Mexico; ^7^ International Potato Center, Maputo, Mozambique; ^8^ International Potato Center, Hanoi, Vietnam; ^9^ National Crops Resources Research Institute (NaCRRI), National Agricultural Research Organization, Entebbe, Uganda; ^10^ North Carolina State University, Raleigh, NC, United States; ^11^ International Development Consulting, Portland, OR, United States

**Keywords:** market segments, breeding pipelines, poverty alleviation; nutrition; gender, investment cases, potato and sweetpotato

## Abstract

Crop breeding programs have often focused on the release of new varieties that target yield improvement to achieve food security and reduce poverty. While continued investments in this objective are justified, there is a need for breeding programs to be increasingly more demand-driven and responsive to the changing customer preferences and population dynamics. This paper analyses the responsiveness of global potato and sweetpotato breeding programs pursued by the International Potato Center (CIP) and its partners to three major development indicators: poverty, malnutrition and gender. The study followed a seed product market segmentation blueprint developed by the Excellence in Breeding platform (EiB) to identify, describe, and estimate the sizes of the market segments at subregional levels. We then estimated the potential poverty and nutrition impacts of investments in the respective market segments. Further, we employed the G+ tools involving multidisciplinary workshops to evaluate the gender-responsiveness of the breeding programs. Our analysis reveals that future investments in breeding programs will achieve greater impacts by developing varieties for market segments and pipelines that have more poor rural people, high stunting rates among children, anemia prevalence among women of reproductive age, and where there is high vitamin A deficiency. In addition, breeding strategies that reduce gender inequality and enhance appropriate change of gender roles (hence gender transformative) are also required.

## Introduction

A recent analysis of the returns to investment by Alston et al. (2020) reveals a healthy scorecard for the Consultative Group on International Agricultural Research (CGIAR). It demonstrates that every United States dollar (USD) invested in CGIAR yields a return of 10 USD, a tenfold benefit. For this impact to be sustained, the CGIAR breeding programs of the Genetic Innovations action area - one of three research action areas under the CGIAR - will need to develop and deploy varieties that meet the needs of breeding customers. Recent evidence suggests that the CGIAR breeding program has tended to be out of sync with the needs and preferences of its customers, resulting in low adoption of new varieties and the disappointingly low turnover of crop varieties produced by the CGIAR and its partners (Walker et al., 2014; Atlin et al., 2017; Thiele et al., 2021). The previously strong demand, reflected by adoption rates as high as 90%, that met the green revolution varieties of the 1960s and 1970s has been replaced by an adoption ceiling of just 40% for some crops (McEwan et al., 2021). In addition, farmers who are the immediate and key customers of the breeding programs often lack information about the available varieties and are hence less inclined to take the risk of switching to new ones. Another important aspect hampering the adoption of new varieties is the lack of planting materials (Thiele et al., 2021).

Potato and sweetpotato are generally highly resilient crops with robust adaptability, short production period, high yield capacity even in marginal lands, and significant nutrition contribution. They form a significant part of cropping systems in areas with high prevalence of poverty, hunger and malnutrition, and contribute immensely to food security and livelihoods of many smallholder farm households in developing countries (Devaux et al., 2020; Kim et al., 2018; Kwak, 2019). Potato is the third most produced food crop globally, after wheat, rice, and maize (FAO, 2020). About two-thirds of the harvest is used for human consumption while the rest are used for seed and animal feed (Devaux et al., 2020). Sweetpotato, on the other hand, is produced in 114 countries and the seventh most consumed staple crop globally (Truong et al., 2018). It is among the top five most important food crops in half of the developing countries (Mukhopadhyay et al., 2011; Kwak, 2019).

To address the improved variety adoption bottlenecks and modernize breeding, the CGIAR and its funders set up the Excellence in Breeding (EiB) platform. The platform aims to increase the rate of variety turnover and specifically to deliver significant genetic gains in farmers’ fields by harnessing a combination of modern breeding techniques and better targeting of varieties to customer needs. One of the key activities implemented by EiB under the Crops to End Hunger initiative (CtEH) was the commissioning of a CGIAR system-wide exercise to develop crop-specific breeding pipeline investment cases (EiB, 2020). The purpose was to understand the current level of investment and the potential impact in the different market segments the programs were targeting. As a start, center breeding programs identified the existing breeding pipelines and defined market segments for mandate crops focusing primarily on poverty alleviation as a system-level outcome. However, the demands on crop breeding programs have, since the green revolution, become more complex and extend beyond meeting food availability/access goals of the 1960s and 1970s when there was a similar need and urgency to modernize breeding programs. Back then, the focus was on yield enhancement to supply sufficient foods for a rapidly increasing population in Asia through breeding for superior genetics, and resistance to pests and diseases (Hazell, 2009).

With the changing demographics, lifestyle and general market needs, the present-day breeding programs have to address the other pillars of food security – especially nutrition – while ensuring gender equity, among other factors. Thus, given the continued expansion of public crop breeding programs globally - and focusing on two mandate crops of the International Potato Center (CIP) - this paper seeks to answer the question: how well targeted are CIP-led potato and sweetpotato breeding efforts towards delivering the greatest impact? It specifically aims to achieve three objectives: i) to describe the interdisciplinary process followed by CIP and EiB in defining market segments and developing breeding pipeline investment cases for potato and sweetpotato global breeding programs, focusing on poverty alleviation; ii) extend the analysis to include nutrition and gender; iii) draw lessons from the exercise to inform future efforts to spearhead demand-driven breeding initiatives. Following the EiB (2020) and Covarrubias-Pazaran et al. (2022), we define a market segment as a target population of environments in which a breeding product is grown, as well as descriptions of the target clients and varietal traits that are valued for production, marketing and consumption by farmers and end-users. A breeding pipeline, in turn, refers to the sum of the research efforts focused on a given market segment or a group of market segments. A breeding pipeline usually will have a clear product handed/advanced at the end of the pipeline and a well-defined customer (Covarrubias-Pazaran et al., 2022). Detailed descriptions of the relationship between product profiles, market segments, and management of breeding pipelines for optimal returns are documented in Cobb et al. (2019) and Ragot et al. (2018). Public-sector crop breeding has primarily focused on tackling productivity challenges to ensure food availability and access, and on improving farmer income (Ceballos et al., 2021). However, since 2010 breeding efforts have shifted to producing nutritionally enhanced biofortified crop varieties (Low et al., 2017). This shift led to intensified investment in breeding for biofortified varieties of staple crops (Low et al., 2022). Women play a significant role in producing these crops, especially the vegetatively propagated crops, and have specific varietal traits needs compared to men (Polar and Demont, 2022).

This paper focuses on analyses of market segments aligned to CIP’s breeding pipelines for potato and sweetpotato focusing on productivity, nutrition and gender. The need to align breeding pipelines with market segments is informed by Hunt and Arnett’s (2004) argument that investments must be designed to efficiently yield commensurate financial returns to the resources spent in each segment. Further, Orr (2018) argues that: i) correct identification of markets has been responsible for the success of hybrid pearl millet dual-purpose cowpea breeding programs and ii) uncertain and misunderstood market conditions lead to low adoption of improved varieties. To make our analysis tractable, we abstract from the analysis of investment costs targeting improvement of specific traits. Readers interested in this aspect can find a good treatment of it in Pemsl et al. (2022). Our focus on productivity, nutrition and gender is driven by a recent literature that argues that crop variety adoption is partially driven by the broader system/ecology in which the breeding and demand are situated^1^ in (see for instance Glover et al. (2016) and Mausch et al., 2021). Thiele et al. (2021) further argue that demand for vegetatively propagated crops, to which potato and sweetpotato belong, has been greatly hampered by lack of focus on quality/sensory traits. Moreover, Mudege et al. (2018) and Gilligan et al. (2020), on the other hand, argue that gender plays key role in scaling up demand/adoption for crop varieties.

The rest of the paper is organized as follows: Section 2 documents the methods used by CIP and EiB in defining market segments and in deriving the investment levels for the breeding pipelines as well as the data sources. In Section 3, we present the results by market segment and investment pipelines for alleviation of poverty and malnutrition and mainstreaming of gender indicators. Conclusions and lessons for policy and practice are discussed in Section 4.

## Materials and methods

Following a series of stakeholder consultations, the EiB developed a blueprint that provided a standard structure of the process, data compilation, and description of the market segments including their sizes and the opportunities within them. This was shared internally within the CGIAR.

This paper draws from findings of a systematic process of a series of consultations among the research team (CIP research leadership, breeding program management team, gender scientists, and led by three socio-economists), a desk review of published and grey literature, key informant interviews, and a series of multi-functional/stakeholder workshops involving breeders, socio-economists, nutritionists, gender scientists, value chain specialists, processors, traders, and breeding program managers. The desk reviews involved gathering data from global open-source databases and exploratory review of crop-specific literature on breeding programs, seed systems, and product market to validate the data and results of the analysis. The key informants consisted of CIP scientists in different disciplines (breeders, gender specialists and agricultural economists), potato and sweetpotato breeding program managers (at CIP and partnering NARS) and the CIP finance team who were useful in, among others, estimating acreages and providing investment levels for each breeding pipeline.

The study was conducted in two phases. Phase 1 focused on identifying and describing market segments, aligning breeding pipelines with the market segments, and estimating and documenting the pipeline investment cases with rural poverty alleviation as the outcome variable. Phase 2 extended the analysis to include assess gender-responsiveness of current breeding efforts and nutrition outcomes. Below, we present details of the steps followed.

### Step 1: Identification and description of the market segments at OneCGIAR sub-regional levels

First, the breeders and socio-economists used the key product differentiating elements for each crop to identify and describe the market segments at regional levels qualitatively and quantitatively. The qualitative part involved assessing: i) The region/location characteristics including the OneCGIAR sub-regions, agroecological zones, country, and target populations environments (TPE). The TPE defines a set of farms and future seasons in which the varieties produced by a breeding program will be grown; ii) The grower parameters including production system (e.g., irrigated versus rainfed), the input level (e.g., small scale versus large scale), and maturity (e.g., early versus late maturing); iii) The consumer parameters such as cooking time, nutritional enhancement (biofortified or otherwise), flesh color (orange, white, or purple), mealiness, hardness and use description (fresh market, home consumption, or for processing).

The quantitative component of the market segment descriptions involved estimation of the size of each market segment and the opportunities for the three outcomes (poverty alleviation, nutrition, and gender equity). Data were gathered from open-access databases including the FAOSTAT^2^, the World Bank^3^, and the Rural Livelihood Information System (RuLIS) ^4^ and included: crop area, total population, rural population, the prevalence of undernourishment, and prevalence of iron anemia among women of reproductive age from the FAOSTAT database – all at country level. The available national datasets do not differentiate products within the countries according to the identified market segment descriptions, hence the use of FAO data.

For poverty^5^ status defined as the national headcount ratio of people living on <1.9 dollars a day, data were drawn from the World Bank database and corrected for 2011 purchasing power parity. We used the rural poverty index given that agriculture is predominantly practiced in rural^6^ areas in the study countries.

Data on hectares targeted for the various breeding products and the yield levels under farmer field conditions in each of the countries covered in a market segment were obtained through consultations with breeders. We then used their expert estimates to break down the data at national levels and align them with the identified market segments. For instance, we used expert estimates of the total hectares under orange, white (including cream and yellow as other shades of white), and purple-fleshed sweetpotato in each country to break down the FAOSTAT data on total population, rural population, rural population under poverty, and undernourished population to the respective sweetpotato market segments in the given country. Further, we consolidated the breakdowns matching the descriptions of market segments from different countries to estimate the size of the market segment at OneCGIAR sub-regional levels.

### Step 2: Aligning the market segments with the breeding pipelines

The CIP breeding unit identified nine breeding pipelines that received CGIAR funding in 2020, the focal year for our analysis. The pipelines focus on key breeding needs/constraints in a specific sub-region. We matched the identified market segments with these breeding pipelines. That is, aligned the market segments with the breeding pipelines that serve the specific crop, subregion, and focus on breeding needs described in the respective market segments. In some cases, the breeding need/constraint spanned more than one region; hence, the pipeline can cover multiple regions, as is the case with table and processing potatoes in Asia.

### Step 3: Estimation of the pipeline investment cases

The estimates of the investments made in the breeding pipelines in 2020 were obtained from the CIP finance team. These were broken down into fixed and variable expenses for each pipeline. Fixed costs included infrastructure, permanent staff salaries/wages, lease/rentals, and institutional costs (including overhead/depreciation). On the other hand, variable costs cover expenses on consumables, temporary staff, genotyping, phenotyping, germplasm improvement, seed production, shipping and other operating costs. We used these and the other data described above to generate pipeline investment cases, that is, estimate the opportunities for breeding to target reduction in rural poverty.

### Step 4: Extension of analysis to include nutrition outcome

While Phase 1 of the analysis considered only rural poverty alleviation potentials, CIP breeding programs have also aimed at addressing nutrition and promoting gender equality. To address the former, we focused on nutrition indicators most relevant to CIP breeding programs where data was available. As in the poverty alleviation case, we used data from FAOSTAT, WHO, and UNICEF focusing on four nutrition indicators: prevalence of stunting in children under-5 years old; prevalence of undernourishment; anemia among women of reproductive age (i.e., 15– 49 years); and the 2-dose vitamin A supplementation (VAS) coverage among children under-5- in each market segment. Despite their relative importance, many nutritional indicators such as zinc deficiency, wasting, underweight and breastfeeding, were not considered due to lack of consistent and reliable country-level data.

Stunting, defined as low height-for-age, is an indicator of chronic malnutrition and results from prolonged food deprivation (de Onis et al., 2018). Prevalence of stunting was estimated by the number and percentage of children under-5 stunted in a market segment. We measured prevalence of undernutrition as the proportion of the population in a market segment whose food consumption is insufficient to provide the dietary energy levels required to maintain a normal, active and healthy life^7^. The VAS, a share of children under-5 receiving vitamin A supplementation, was measured as a three-year (2018-2020) average of the VAS coverage. Prevalence of anemia, lack of iron, was measured by the number and share of women of age 15-49 years who are deficient in iron.

The country-level data on the selected nutrition indicators were disaggregated to the market segment level using expert estimates of the land area covered by the crops described in the respective market segments. These were further corrected for rural population density data based on the premise that a majority of farming activities in the developing world are mainly rural based (Rapsomanikis, 2015), and that a high proportion of the malnourished is also more likely to be found in the rural areas (Micha et al., 2020).

### Step 5: Gender analysis of the market segments and pipelines

We used the G+ tools^8^ (G+ Customer Profile and G+ Product Profile tools) to assess the gender responsiveness of the customer and product profiles of CIP’s sweetpotato and potato breeding pipelines. The tools are designed to help breeders use a gender lens in characterizing customer segments and evaluating breeding traits, that is, mainstream gender into trait selection (Ashby and Polar, 2021). The tools have been piloted in multiple countries and crops, including cassava in Nigeria and sweetpotato in Uganda (Polar and Demont, 2022).

Using the G+ Customer profile tool, we mapped the customers for the various potato and sweetpotato products promoted in respective market segments using gender-disaggregated data from peer-reviewed and grey literature. We then engaged the multifunctional teams (including breeders, agronomists, socio-economists, nutritionists, gender scientists, value chain specialists, processors, traders, and farmers operating in the respective subregions), from CIP and related NARS, to validate the characterization of the customers by discussing the relative gender roles of the customers, and their needs and preferences concerning the commodity. They also discussed their desired approaches for mainstreaming gender in breeding programs targeting the respective market segments. The two gender mainstreaming approaches discussed include: 1) A transformative approach that advocates for progressive changing of the power structures that motivate unequal gender relations in the target segment. 2) A functional approach that acknowledges and reacts to the existing gender inequality by strategically designing and delivering the breeding products to improve adoption in the respective segments as constituted.

**Figure 1:**
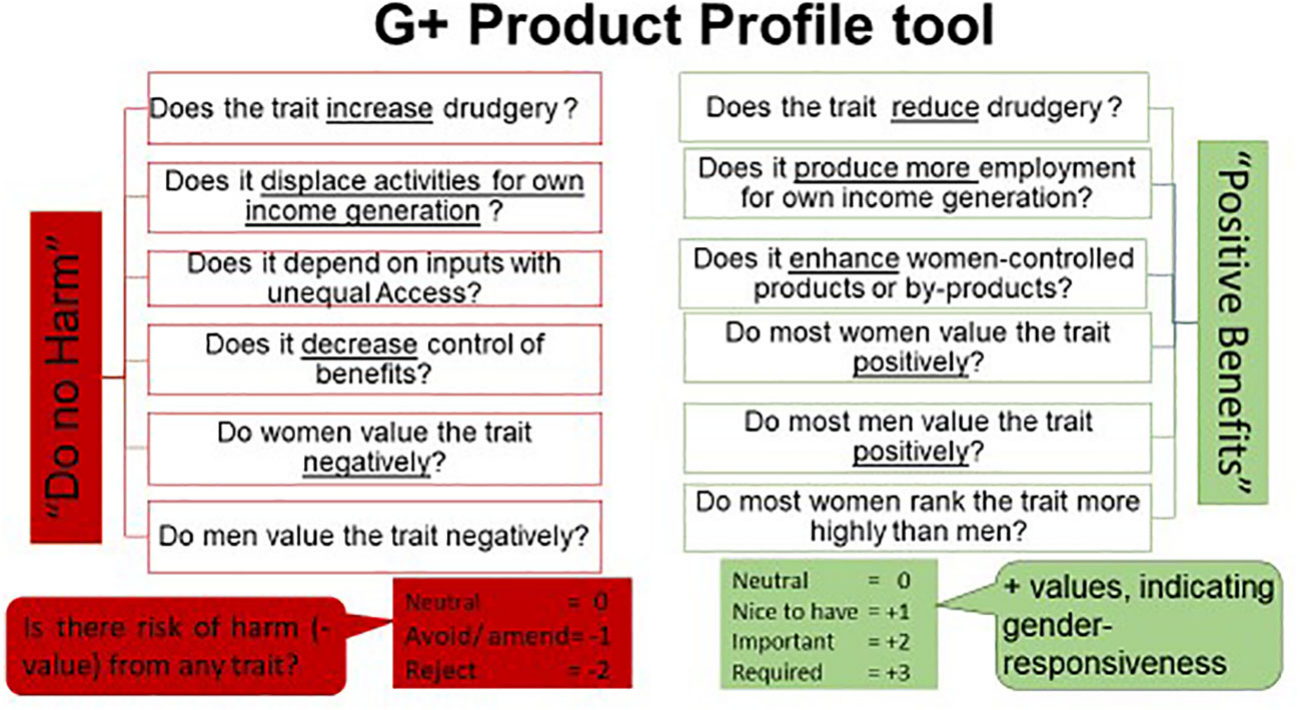
The outline of the G+ product profile query for gender impact scores.

Following the G+ Product Profile tool, we assessed potential negative (harmful) and positive (beneficial) effects that breeding for a specific trait or combination of traits could have on women and men, focusing on four important dimensions of gender equity in agriculture. These dimensions are drudgery and time poverty; control over critical on-farm resources (e.g., land); access to critical purchased inputs for the crop and product; and control over the sharing of benefits (e.g., crop income). **Figure 1** presents the tool’s framework for querying and scoring these dimensions. After considering these dimensions for each trait proposed for a product profile, a gender-responsive breeding program should at a minimum, seek to have a neutral “*do no harm*” and ‘*positive benefit*’ score by ensuring that the trait or combination of traits pursued does not make women worse off (Ashby and Polar, 2021).

The “*do no harm*” assessment thus aims to minimize the risk of releasing a variety that could increase gender inequity and culminates in assigning each trait a qualitative gender impact score namely: 1) “Neutral”– implying the trait causes no significant harm to a majority of the women; 2) “Amend”– the trait could increase the gender inequity that would need to be mitigated/amended during the delivery/dissemination process; 3) “Reject”– the trait causes significant harm to a majority of one of the genders and should be dropped from the product profile.

The “positive benefit” assessment aims to flag the positive contribution of the traits to the reduction in gender inequity. It ends with assigning traits scores, at 4 levels, which reflect its benefit to women and men, namely: 1) “Neutral,” – implies that the trait induces no issue related to gender equity. 2) “Nice to have” – women and/or men derive some advantage from this trait, but they can do without it. 3) “Important” – the trait confers positive benefits to men and women, making it indispensable. 4) “Required” – the trait is of high value to a majority of women, and it is essential to enhancing gender equity.

**Figure 2:**
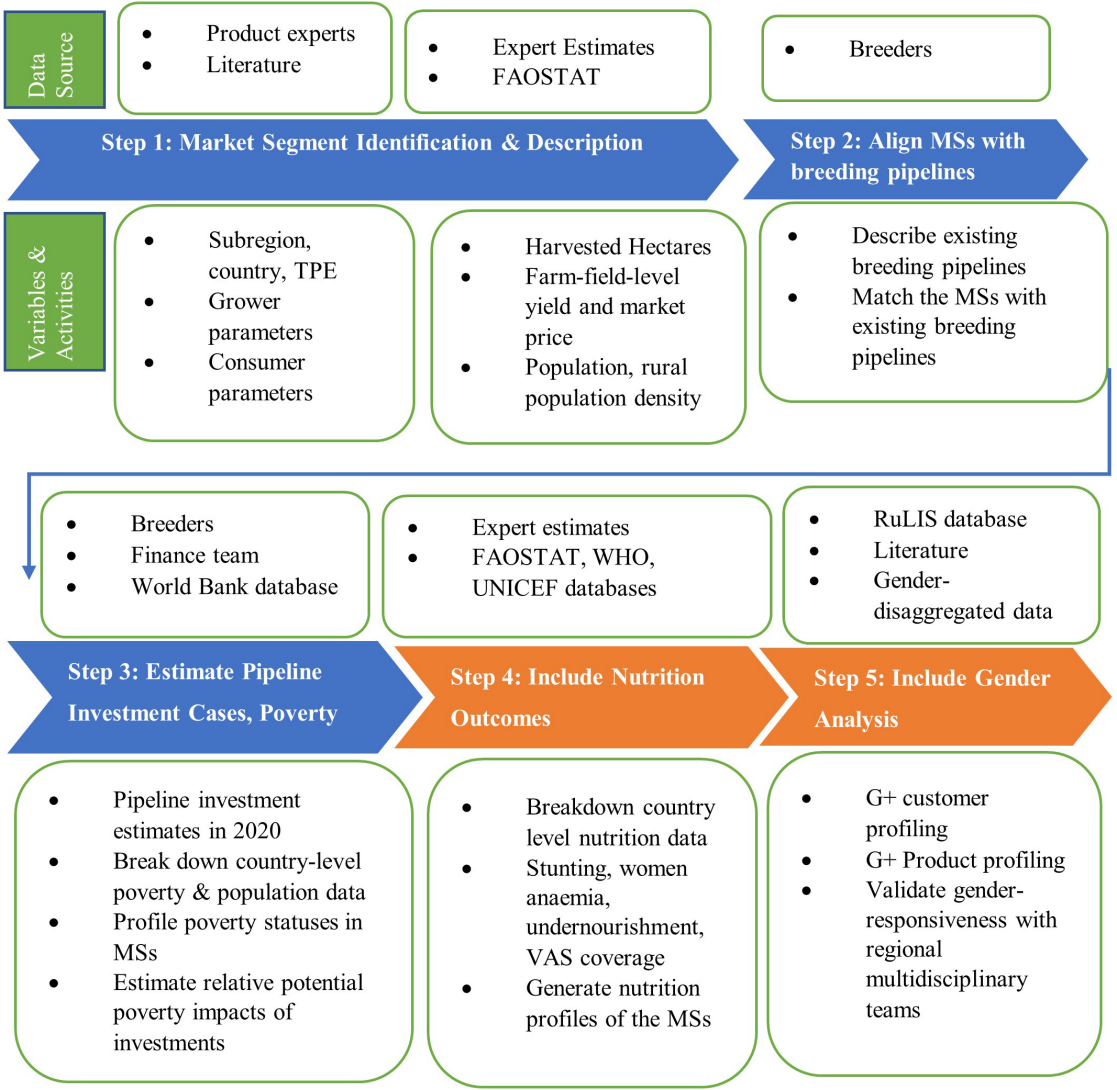
A graphical summary of the study methodology.

The “do no harm” and “positive benefit” assessments require evidence-based judgments and choices by experts who do the scoring. Therefore, as recommended by Orr et al. (2021) and Ashby and Polar (2021), for each region, we convened in-person and virtual multidisciplinary cross-functional teams comprised of social scientists, breeders, food scientists, and other relevant value chain actors to assess/assign gender impact scores for different product profiles, separately, to validate them for contextual relevance. Separate teams were constituted for each region. Each team assessed the customer and product profiles for their respective regions. The regions covered included East Africa, Southern Africa, West Africa, and Latin America and the Caribbean (LAC). **Figure 2** presents a graphical summary of the study methodology.

### Description of market segments

We identified twenty-three market segments that the CIP breeding program targets: 14 for sweetpotato (**Table 1**) and 9 for potato (**Table 2**). The key elements distinguishing the market segment are sub-region and flesh color (white/cream, orange, or purple) for sweetpotato, and sub-region and use description (table, processing, or dual purpose) for potato. For both crops, and in consultation with breeders, we identified 7 sub-regions namely: East Africa (EA), Southern Africa (SNA), West Africa (WA), Latin America and the Caribbean (LAC), Central Asia (CA), South Asia (SA) and South-East Asia (SEA). In addition, we considered differences in the agroecologies within and across regions in describing the market segments. For instance, the Southern African one is characterized by drought-prevalence, irrigation status (irrigated *vs* rain-fed), and length of the season (short) while the EA sub-region is characterized by high sweetpotato virus disease (SPVD). The resulting market segments for sweetpotato are white-fleshed sweetpotato (WFSP) for home consumption and fresh markets; orange-fleshed sweetpotato (OFSP) for home consumption, fresh markets and local processing; and purple-fleshed sweetpotato (PFSP) for home consumption and fresh markets. The WA sub-region has two key agroecologies each with WFSP and OFSP market segments. These are the: 1) rain-fed long-season tropical zone, and 2) drought-prone Savannah and Sahel zone with a short season, irrigated and rain-fed systems. The LAC, CA, and SA sub-regions each have one market segment, i.e., OFSP while SEA has one PFSP segment.

**Table 1:** Hectares targeted in the sweetpotato market segments.

Region	East and Southern Africa							West and Central Africa					LAC		CWANA		South Asia		SEAP	
Sub-region	East Africa			Southern Africa				West Africa					LAC		Central Asia		South Asia		South East Asia	
									Tropical zone		Savannah and Sahel									
Country	WFSP	OFSP	PFSP	Country	WFSP	OFSP	PFSP	Country	WFSP	OFSP	WFSP	OFSP	Country	OFSP	Country	OFSP	Country	OFSP	Country	PFSP
Tanzania	500	90	20	Malawi	450	90	15	Nigeria	108	11	60	5	Brazil	60	Tajikistan	8	Bangladesh	26	Vietnam	23
Uganda	450	80	20	Madagascar	130	30	8	Ghana	60	5	8	2	Peru	17	Uzbekistan	4	India	131	Indonesia	23
Rwanda	180	50	10	Mozambique	120	20	7	Cote d’Ivore	18	3	7	2	Haiti	10			Pakistan	6	Philippines	21
Eastern DRC	110	20	7	Zambia	50	10	5	Sierra Leone	18	3										
Burundi	90	20	5	Zimbabwe	15	10	3	Guinea	19	2										
Kenya	80	20	5					Benin	9	2										
Ethiopia	60	15	5					Liberia	2.8	1										
								Mali			18	3								
								Burkina Faso			6	2								
								Niger			6	1								
**Total**	**1,470**	**295**	**72**		**765**	**160**	**38**		**234.8**	**27**	**105**	**15**		**87**		**12**		**163**		**67**

Values are in thousands of hectares. LAC, Latin America and the Caribbean; CWANA, Central and West Asia and Northern Africa; SEAP, South East Asia and the Pacific; WFSP, White-fleshed Sweetpotato; OFSP - Orange-fleshed Sweetpotato; PFSP, Purple-fleshed Sweetpotato.

**Table 2:** Hectares targeted in the potato market segments.

Region	East and Southern Africa		LAC			South Asia				SEAP		
Sub-region	East Africa		LAC			South Asia				South East Asia		
							Lowlands	Highlands			Highlands	
Country	Table	Dual-purpose	Country	Table	Dual-purpose	Country	Table	Table	Processing	Country	Table	Processing
Kenya	200	50	Peru	334	330	India	217	2,150	220	Indonesia	68	7
Rwanda	104	26	Bolivia	135	200	Bangladesh	50	477	215	Vietnam	24	3
Ethiopia	72	8	Colombia	125	130	Myanmar	5	33	3			
Uganda	36	4	Venezuela		30	Vietnam	0.5					
			Ecuador	52	24							
**Total**	**412**	**88**		**646**	**714**		**272.5**	**2,660**			**92**	**10**

Values are in thousands of hectares. LAC, Latin America and the Caribbean; SEAP, South East Asia and the Pacific.

For potato, both EA and LAC regions have two market segments defined by potato use as table versus dual-purpose (suitable for both processing and table) (**Table 2**). In the EA segments, fast cooking, early maturity and short dormancy are the defining traits for the potato grown, while in LAC, medium maturity and biofortification/nutrient density are the desired traits. Still, for potato, SA and SEA sub-regions each have 2 common potato market segments: a market for high dry matter, early maturing, yellow-fleshed table potato; and another market for high dry matter, late blight resistant, yellow-fleshed processing potato. In addition, the South Asian lowlands is another market segment, which demands early maturing, salinity, heat and drought-tolerant table potato.

## Results

### Market segment sizes and opportunities for poverty alleviation impacts

Starting with sweetpotato, **Table 3**, the East African market segments have the highest targeted hectares, the value of the production, total population, and population in poverty aligned with them compared to other regions. Central Asia, West Africa’s Savannah and South-East Asia (SEA) have the lowest number of poor people, total population and hectares targeted for sweetpotato, respectively. The white-fleshed sweetpotato market segments dominate in three regions in Africa in terms of hectares under production, whereas the OFSP is the only type of sweetpotato bred for the LAC, Central Asia and South Asia market segments. The purple-fleshed sweetpotato varieties are bred for the Southeast Asia region.

**Table 3:** Size of sweetpotato market segments and potential poverty impact in the pipelines.

Sub-region	East Africa			Southern Africa			West Africa - Tropical zone		West Africa -Savannah and Sahel zone		LAC	Central Asia	South Asia	South East Asia
Market Segment (MS)	WFSP	OFSP	PFSP	WFSP	OFSP	PFSP	WFSP	OFSP	OFSP	OFSP	OFSP	OFSP	OFSP	PFSP
Value of harvest (million $US)	3,675	1,062	216	1,913	576	114	587	81	210	45	313	40	538	482
# of people in MS	3,411,845	747,963	176,013	1,408,968	287,187	53,108	192,254	23,298	46,095	6,834	39,368	13,718	970,154	257,838
# of poor people in MS	1,762,419	390,779	91,571	981,953	197,357	35,934	66,693	8,564	18,234	2,693	7,203	2,093	179,117	9,587
Pipeline Investments^*^	34.49%			47.08%			None in 2020		None in 2020		6.95%	6.31%		5.18%
Rank of investment per a million-dollar harvest**^†^**	4			5			–		–		3	2		1
Rank of $US per person**^‡^**	3			2			–		–		1	5		4
Rank of $US per person in poverty**^‡^**	4			3			–		–		1	5		2

* Pipeline investments are presented as a percentage of total investment estimates committed to sweetpotato breeding pipelines in 2020.

† A pipeline with the lowest investment estimates per a million-dollar worth of harvest is ranked #1 while the pipeline with the highest investment estimates per a million-dollar worth of harvest is ranked #5.

‡ A pipeline with the highest investment estimates per person or person in poverty is ranked #1 and that with the lowest investment per person or person in poverty is ranked #5.

Turning to potato, **Table 4**, the South Asian market segment for yellow-fleshed, high dry matter, early maturing table potato has the highest population, value of production, number of poor people living in poverty, and hectares targeted while the Southeast Asian market segment for high dry matter, late blight resistant (LBR), yellow-fleshed processing potato has the lowest.

**Table 4:** Size of potato market segments and potential poverty impact in the pipelines.

	East Africa		LAC		South Asia Lowlands	South Asia Highlands	South East Asia	South Asia	South East Asia
Market Segment (MS)	Table	Dual	Dual	Table	Table	Table	Table	Processing	Processing
Value of harvest in million $US	1,813	387	2,142	1,938	736	10,534	364	643	27
# of people in MS	1,111,748	243,408	177,904	177,904	1,669,363	16,255,257	256,552	1,274,547	29,099
# of poor people in MS	509,529	116,270	5,977	6,177	308,051	3,017,424	7,163	251,315	788
Pipelines investments*	26.27%		9.59%	16.98%	7.85%	27.18%		12.13%	
Rank of investment per a million-dollar harvest^†^	5		2	3	4	1		6	
Rank of $US per person^‡^	3		2	1	5	6		4	
Rank of $US per person in poverty^‡^	4		2	1	5	6		3	

Dual, Dual purpose i.e., the potato can be used for both processing and table; MS, market segment.

* Pipeline investments are presented as a percentage of total investment estimates committed to potato breeding pipelines in 2020.

† A pipeline with the lowest investment estimates per million-dollar worth of harvest is ranked #1 while the pipeline with the highest investment estimates per million-dollar worth of harvest is ranked #6.

‡ A pipeline with the highest investment estimates per person or person in poverty is ranked #1 and that with the lowest investment per person or person in poverty is ranked #6.

The results also show that the 2020 sweetpotato pipeline investments were highest in the Southern Africa segments, taking about half of the total investment in sweetpotato breeding efforts in the same year (**Table 3**). There was a higher investment per person living in poverty in Southern Africa than in East Africa. In addition, for both crops, the LAC region had the highest estimates of investment per person living in poverty– ranked number 1 in sweetpotato (**Table 3**), and 1 and 2 in the potato crop (**Table 4**).

### Nutrition profiles and potential nutritional impacts of investment in the market segments

**Table 5** presents the nutrition profiles of the sweetpotato market segments and pipelines. The stunting rate among children under 5 years in the East African sweetpotato pipeline is 35.7% amounting to 20.9 million children. The majority of the stunted are in the WFSP segment (18.2 million). Across the board, the number of stunted children aligned with the WFSP market segments is higher than for those aligned with the OFSP market segments: 5.5, 4.5, and 6.7 times higher in East Africa, Southern Africa and West Africa pipelines, respectively. The OFSP segment targeting Central Asia and South Asia has the highest number of stunted children (43.7 million), followed by East Africa (20.9 million) and West Africa pipelines (19 million).

**Table 5:** Nutrition status in the sweetpotato market segments.

Sub-region	East Africa				Southern Africa				West Africa Tropical		West Africa Savannah and Sahel			LAC (P3)	Central Asia	South Asia		SE Asia P5
Market Segment and pipelines (P#)	WFSP	OFSP	PFSP	P1	WFSP	OFSP	PFSP	P2	WFSP	OFSP	WFSP	OFSP	PW^†^	OFSP	OFSP	OFSP	P4	PFSP
Stunted children *	18.2	3.3	0.8	20.9	4.9	1.1	0.2	6.2	7.4	1.1	9.4	1.1	19	1.5	0.6	43.1	43.7	12.6
Stunted children (%)	35.7	35.7	35.7	35.7	34.8	34.8	34.8	34.8	28.2	28.2	27	27	27.6	12.8	13.2	29.8	23.1	28.0
VAS coverage (%)	60.8	60.8	60.8	60.8	58.1	58.1	58.1	58.1	62.8	62.8	54	54	58.4	20.3	91.3	69.4	74.9	29
Women Anemia*	23.3	4.6	1.1	28.5	7.7	1.7	0.4	9.8	15.2	2.3	22.5	2.7	42.7	12.4	3	208.9	211.9	30.7
Women anemia (%)	31.6	31.6	31.6	31.6	35.4	35.4	35.4	35.4	50.5	50.5	48	48	49.3	28.1	29.9	43.8	38.2	21.2
# Undernourished*	69.7	13.8	3.5	86.3	19.3	4.1	1	24.4	14.6	2.1	24.8	3.2	44.7	4.1	–	228.6	228.6	34.2
% Undernourished	28.6	28.6	28.6	28.6	30.6	30.6	30.6	30.6	12.1	12.1	18.1	18.1	15.1	27.8	2.4	10.9	10.9	7.5
Invested dollars per undernourished person				7.4				37.5					–	23.9			2.2	9.5

*Values are provided in millions. Columns with labels P# (e.g., P1, P2…) present data at pipeline levels. Some pipelines have one market segment e.g., P3 and P5. The other pipelines (P1, P2, P4 and PW) cover multiple market segments presented in the preceding columns within the region(s).

^†^West Africa sweetpotato pipeline (PW) did not receive funding in 2020.

The VAS coverage in the sweetpotato market segments and pipelines is highest in Central Asia and South Asia pipelines (74.9%) and lowest in the LAC pipeline (20.3%). Only the Central Asia and South Asia OFSP segment has an average VAS coverage greater than the WHO-recommended 70% (WHO, 2009). West Africa segments, which did not receive funding commitments in 2020 also have considerably low VAS coverage.

In terms of anemia, the number of women who are anemic is highest in the WFSP followed by OFSP and the PFSP market segments for sweetpotato pipelines in Africa. The West Africa pipeline has the highest prevalence of anemia in women (49.3%) while South-East Asia has the lowest (21.2%).

Turning to potato again (**Table 6**), more stunted children are aligned with the table potato market segments than the dual-purpose or processing market segments in the respective pipelines. For instance, the East Africa potato pipeline has a stunting rate of 29.5%, amounting to 12.7 million stunted children (i.e., 10.7 million and 2 million children in the table and the dual-purpose potato segment, respectively).

**Table 6:** Nutrition status in the potato market segments.

Region	East Africa			LAC		South Asia Lowlands	South Asia Highlands	South East Asia		South Asia	South East Asia	
Market segments and pipelines (P#)	Table	Dual-purpose	P6	Dual (P7)	Table (P8)	Table (P9)	Table	Table	P10	Processing	Processing	P11
Stunted children*	10.7	2	12.7	1.3	1.6	3.5	36	0.5	36.5	3.4	0.1	3.5
Stunted children (%)	29.5	29.5	29.5	15	14.1	29.8	29.8	13.2	23.1	29.8	13.2	23.1
VAS coverage (%)	55	55	55	40.7	40.7	69.4	69.4	91.3	74.9	69.4	91.3	74.9
Women Anemia*	15.9	2.9	18.9	6.1	7.9	23.1	174.3	2.7	177	16.7	0.3	17
Women anemia (%)	30.9	30.9	30.9	20.8	21.4	43.8	43.8	29.9	38.2	43.8	29.9	38.2
# Undernourished*	38	7.7	45.7	10.9	18.7	26	190.7	–	190.7	18.5	–	18.5
% Undernourished	22	22	22	10.6	14	10.9	10.9	2.4	8.8	10.9	2.4	8.8
Investment per undernourished person			3.0	51.5	29.4	1.6			0.6			3.3

*Values are provided in millions. Columns with labels P# (e.g., P6, P7…) present data at pipeline levels. Some pipelines have one market segment e.g., P7, P8 and P9 while the other pipelines (P6, P10, P11) cover multiple market segments presented in the preceding columns within the region(s).

The prevalence of undernourishment is higher in East Africa potato pipelines (22%) than in the other pipelines. However, due to expected differences in population densities, the South Asia and South-East Asia table potato pipeline has the highest number of undernourished people.

The Asian processing potato for tropical South-East Asia has the highest VAS (i.e., 91.3%) and lowest stunting rate (13.2%) among the potato market segments and pipelines considered. It also has the highest anemia prevalence rate (29.8%) when compared to the Andean biofortified table and dual-purpose potato segments (21.4% and 20.8%, respectively).

### Results of the multistakeholder gender profiling of the market segments

**Figure 3:**
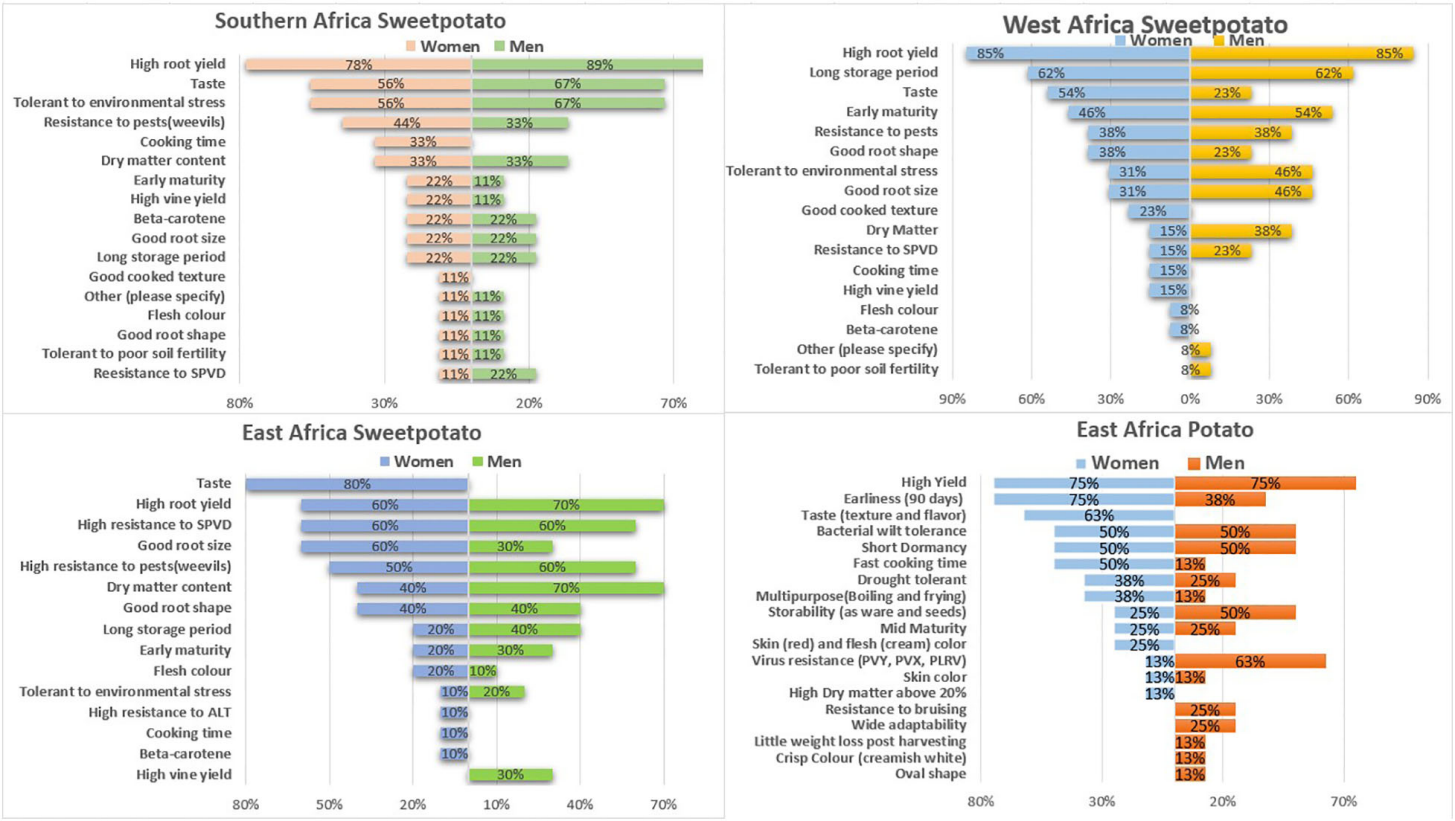
Stakeholders perception of major sweetpotato and potato traits preferred by women and men in the pipelines.

From the customer profile analyses, **Figure 3** presents the key traits preferred by women and men across four pipelines. Some preferred traits– including cooking time, good texture, root size and taste– have gender implications not only because women select the varieties that they consider desirable for household consumption but because women almost always make up the bulk of small-scale processors whose profit margins and income depend on these quality traits.

**Figure 4:**
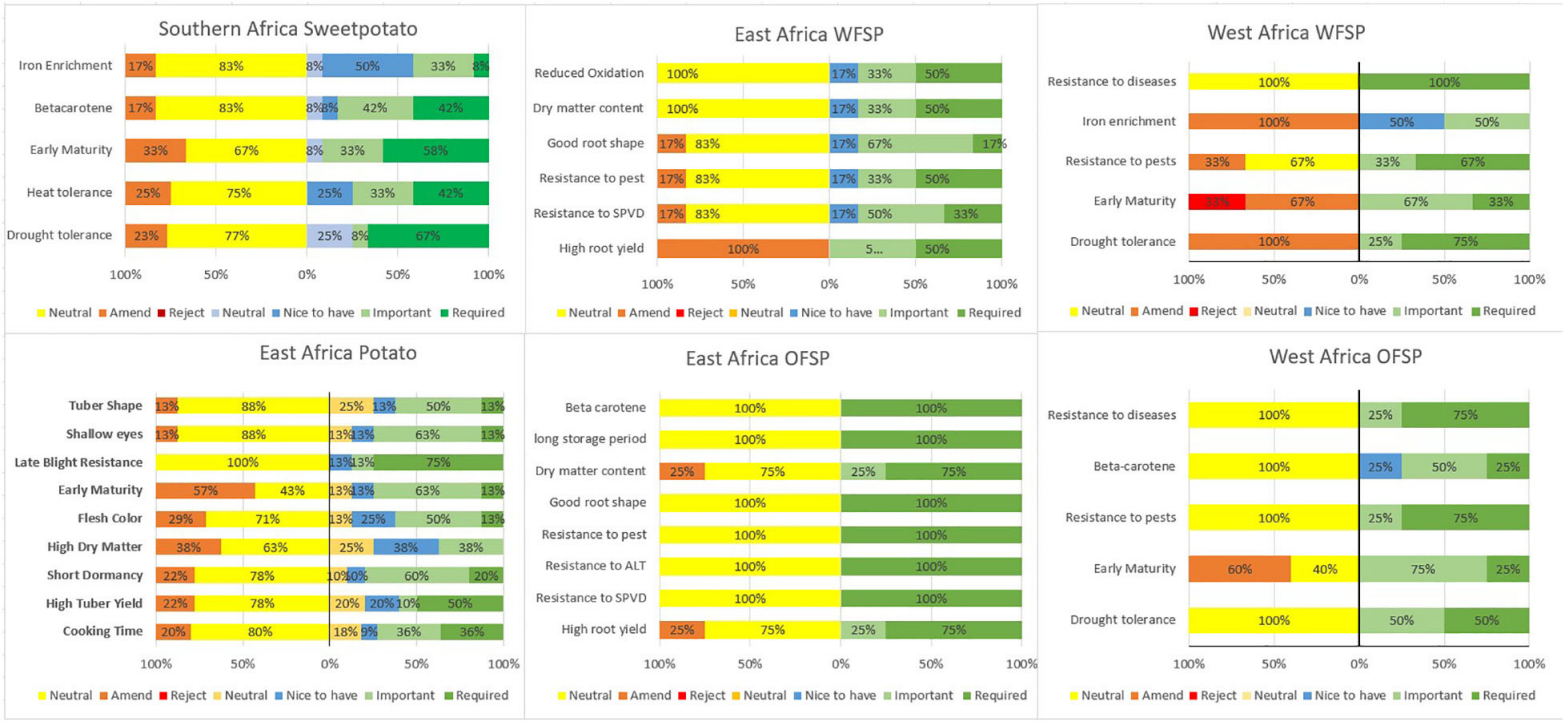
Cross-functional workshop results on gender impact scores of the traits in different potato and sweetpotato pipelines.

**Figure 4** presents the results of the G+ product profile analysis of products bred for 6 select market segments that went through a complete analysis with the G+ tools involving the multifunctional teams. The analyses follow the scoring framework presented in **Figure 1**. The results show that all the breeding pipelines have traits associated with “*do no harm*”. These are traits scored as “*amend*,” flagging the need for non-breeding interventions to mitigate gender-unequal adoption outcomes. For example, early maturity was scored as “*amend*” in all the potato and sweetpotato breeding pipelines for the concern that higher revenues from early season sales of produce previously destined for home consumption and under women’s control, could attract men to take over sales and displace women.

## Discussion

Attempts to replicate the successes of the Asian green revolution through supply-side breeding interventions that primarily focus on developing and releasing new crop varieties in many parts of the developing world have largely failed in recent decades due to changing market needs and population dynamics. Indeed, instituting a demand-driven breeding initiative requires action from a multidimensional approach, and redesigning and/or reprioritization of investment in breeding programs to be more nutrition-sensitive and gender-responsive is not a panacea, but a significant part of the solution. Alongside integrating nutrition and gender objectives in breeding programs, significant investments are needed to ensure that the delivery system encompassing the entire food chain (from farming to nutrition) is at sync with the genetic advancement, and both match the needs of the target market segments. These should also consider the diversity of the respective food systems of the farmers and consumers in the market segments. Nonetheless, breeding for high yielding, nutritious, gender-responsive, more inclusive and climate resilient varieties would offer the greatest impact at a macro level. It is important that we challenge ourselves with the question, how more impactful would the green revolution crops be in Asia if they were not only high yielding but also more nutritious, gender responsive, and climate resilient?

While the twin pursuits of achieving food security and reducing poverty status remain the key focus of most public breeding programs, the need to achieve nutrition security and gender equity has changed the way the successes of breeding programs is measured and hence raised concerns about how best to allocate breeding investments funds among different market segments targeted. This paper presents a systematic analysis of market segments and breeding pipelines pursued by the International Potato Center, a member of the One CGIAR, and its national partners across the globe. It first focuses on the goal of breeding to address food security, hence, tackling rural poverty. It then offers insights on how these segments and pipelines have performed on three key nutrition indicators (stunting among children under 5 years of age, vitamin A supplementation coverage, and anemia prevalence among women of reproductive age), and four gender inequity indicators (drudgery and time poverty, control over on-farm resources, access to external inputs and control over benefit sharing among vulnerable people, especially women).

Our results offer certain key insights. The East African sweetpotato market segments have the highest population, production value, poor people and targeted hectares. On the other hand, the Central Asia sweetpotato segment has the lowest number of poor people while the total population and targeted hectares are the lowest in the West African and the South-East Asian sweetpotato segments, respectively. Within Africa, it is interesting to note that despite the enormous opportunity (indicated by population, a proxy for market potential) in the East Africa sweetpotato breeding pipeline; it received lower investments than the Southern Africa breeding pipeline. This is because of the higher level of private investments in the Southern African breeding pipeline through bilateral arrangements. It highlights the importance of public-private sector partnerships. Similar partnerships are needed to take advantage of the East African pipelin’’s huge market potential/opportunity. For potato, the South Asian segment (with the high dry matter, early maturing, yellow-fleshed table potato) has the highest population, the number of poor’people, and the hectares targeted. On the contrary, the South-East Asian segment (with high dry matter areas with LBR yellow-fleshed processing potato) has the lowest levels for these indicators. At the pipeline level, while Latin America has the highest investment per 1,000 poor people in the region, this investment has sizeable global spillover effects. The Asian table potato for tropical and subtropical areas with LBR has the highest potential impact and lowest investment per poor people.

### Breeding pipeline investments relative to poverty levels

Investment per person living in poverty was higher in the Southern Africa pipeline than in the East Africa pipeline. This could be due to the multiple goals pursued by the Southern Africa breeding platform which include drought tolerance, micronutrient (i.e., iron, zinc and beta carotene) enrichment, and resistance to biotic constraints (namely, pests and diseases) (Keneni et al., 2016; Ranjith and Rao, 2021). In terms of the production gains, about one-half of the dollars invested in sweetpotato breeding in the Southern Africa region would produce one million dollars (hence double) worth of sweetpotato in East Africa.

For both crops, LAC pipelines attracted the highest level of investment relative to the population in poverty since CIP breeding programs assume a centralized approach where many breeding lines start at the headquarters in Peru. Candidate varieties from the headquarters are then sent to other regions for performance trials and release by the national agricultural research systems (NARS). In other situations, the LAC pipelines ship true seeds to NARS programs or the more recently established CIP breeding hubs. In all cases, there is a spillover of efforts from the LAC breeding pipelines to the other pipelines. In addition, the pipeline that serves the South Asian highlands and South-East Asia with table potato has the largest poor population (over 3 million) and the lowest investment per population living in poverty (ranked number 6) (**Table 4**). This result highlights the need for deliberate consideration of the relative population poverty density when apportioning investments and/or evaluating the real poverty impacts of investing in the targeted market segments.

### Potential for nutritional impacts in the market segments and pipelines

Vitamin A deficiency is a significant public health concern in all market segments. However, the suboptimal VAS coverage across the market segments demonstrates its inadequacy in the fight against vitamin A deficiency (VAD). This corroborates the finding by Bhutta et al. (2013) that nutrition-specific interventions alone cannot fully address stunting, hence the need for nutrition-sensitive approaches such as the biofortification of staples. Thus, the lack of funding for the West Africa pipeline is a missed opportunity for a potentially higher impact on VAD alleviation in the region compared to the other regions. The literature indicates that OFSP has great potential for reducing stunting in young children as it facilitates the increased intake of vitamin A (Girard et al., 2014; Girard et al., 2021). Nonetheless, the analysis shows that the stunted population reached by OFSP products is relatively low compared to the dominant WFSP.

The dominance of white-fleshed sweetpotato varieties, which have negligible beta-carotene and iron content coincides with the high malnutrition rates in the regions in sub-Saharan Africa. This alignment demonstrates the level to which nutritional needs of the population remain underserved by CIP breeding and product promotion programs. Nevertheless, relative investment per undernourished person was highest in the Southern Africa pipeline and lowest in Central Asia and South Asia pipeline. This difference can be attributed to the variations in the population densities between the market segments.

Generally, regarding sweetpotato, all nutrition indicators show that more people need nutritional support in the WFSP market segments than in the OFSP and PFSP market segments in Africa. For instance, in West Africa, the WFSP has more stunted children (7.4 million), higher VAS coverage (62.8%), and a higher prevalence of anemia (50.5%) than its OFSP segment (1.1 million stunted children, 54% VAS coverage and 48% anemia prevalence). Therefore, for the sweetpotato pipelines in Africa, greater nutritional impacts can be achieved by investing more in the OFSP market segment.

Turning to potato, we observe mixed results of prevalence of nutritional indicators on the potato market segments. For instance, the low stunting in the East African processing potato segment shows that investment in developing the processing potato has a greater potential impact on reducing stunting by addressing multiple household needs (i.e., food and income). In addition, the lowest stunting rate and highest VAS coverage in the case of Asian processing potato for South-East Asia provide an opportunity to learn from in addressing high anemia prevalence in East Africa and the Andean regions.

The VAS coverage is higher in the potato pipelines in Asia (69.4% in the South-East Asia lowlands table potato, 74.9% in the South Asia highlands table and processing potato pipelines) and lowest in the LAC pipelines. However, the Asian table potato pipelines have the highest (43.8%) anemia prevalence implying a greater need for iron-deficiency alleviating interventions in this market segment and indicating the opportunity for impact from breeding iron-rich potatoes. Also, both potato market segments in East Africa pipeline have a relatively higher anemia prevalence of 30.9% than those in the LAC and the tropical South-East Asia potato pipelines. Recent literature shows that there is an opportunity for breeding programs to introduce iron biofortified potatoes in these segments to combat anemia (Amoros et al., 2020) given the finding that iron absorption from iron-rich potatoes is exceptionally high (Jongstra et al., 2020).

### Mainstreaming gender in breeding for the market segments

From the G+ customer profile analyses, involving the multistakeholder teams, it was observed that the existing product profiles for the different potato and sweetpotato market segments missed one or more of the key traits preferred by women in the respective regions. A case in point is where taste was missing in the East Africa potato product profiles, but 80% of stakeholders confirmed that it is exclusively a top trait for women. Taste also features for the Southern and West Africa sweetpotato segments but is not one of the targeted value-added traits. The customer profiling also indicates that some prioritized traits in existing product profiles are not key per the preference list e.g., oxidation was not mentioned in the sweetpotato segment while beta-carotene had minority mentions, implying that stakeholders are not widely familiar with desirable traits of improved varieties.

Further, while evaluating the gender responsiveness of the product profiles, the stakeholders identified a trade-off between the positive and negative impact of high yield when the majority voted *“amend”* for the ‘high yield’ variety trait in the East and West Africa potato pipelines. They associated higher yield with the increased burden of unpaid family labor for harvesting and cleaning the tubers for the market, much of which is often supplied by women and children. This assessment is in line with research showing that men appropriate increased returns from the commercialization of crops without remunerating other family members (Sinelle, 2018). We further found that in potato production, in line with Mudege et al. (2018), men tend to focus more on marketing, further corroborating the concern with the displacement of women when the crop becomes commercialized. High dry matter content was scored as “*amend*” in both potato and sweetpotato, but the level of dry matter preferred in potatoes varied with the intended use: low dry matter for boiling and medium dry matter for crisping.

The abiotic and biotic risk-related traits were associated mainly with positive benefits from a gender perspective. These include tolerance to environmental stresses in Southern Africa; high resistance to the sweetpotato virus disease (SPVD) in East and West Africa; and resistance to late blight disease (LBD) in potatoes in East Africa, which causes between 30% – 60% losses to more than 50% of the farmers (Nyankanga et al., 2004; Otieno, 2019). These traits translate to reduced need for and effort towards irrigating the crops and managing pests and disease infections. Related activities such as fetching water for irrigation and increased rounds of weeding/earthing up would increase drudgery among women.

Overall, this analysis suggests that strengthening gender responsiveness of the breeding pipelines will require the varieties bred in the future to take account of gendered quality traits, taste in particular, where these are missing from current product profiles, in ways that promote women’s income generation. In addition, the “*do no harm*” analysis flagged the need for varietal dissemination strategies intentionally designed to change gender relations positively to mitigate unequal benefit sharing, and/or displacement of women due to market potentials as several studies have documented (Njuki et al., 2011; Rao and Huggins, 2017; Dzanku et al., 2021).

A majority of cross-functional teams from East, West, and Southern Africa (70%, 71.4%, and 100%, respectively) voted in favor of pursuing a functional approach to mainstreaming gender in the breeding programs. This opinion supports the design of new products (i.e., varieties) that avoid handicaps to adoption due to existing and changing gender relations in the target market segments. The stakeholders noted that breeding initiatives have limited potential for introducing significant changes in a crop’s gender roles, responsibilities and relations but can stimulate complementary gender-responsive innovations. For instance, extension services could promote staggered planting and piecemeal harvesting of an early maturing plant to mitigate the burden of labor and associated time poverty among women. The stakeholders assessing gender responsiveness of potato traits in the LAC region, however, voted for a gender transformative approach to breeding (66.7%). That is, delivering new products (varieties) that cause changes in gender roles and responsibilities such as the development of varieties that foster increased involvement of women in activities that are or can be dominated by women (e.g., large-scale marketing by women’s cooperatives). In essence, all but one of the cross-functional scoring teams decided that the breeding pipelines assessed cannot re-design varieties to change gender inequality in commercialization but can reach out to other service providers and forewarn them of the need to complement new varieties with other gender-responsive innovations. This can ensure attention to key preferred traits and the design of gender-responsive interventions that reduce gender inequalities exacerbated by new traits. Moreover, it is important to involve multidisciplinary stakeholders in defining quality and market traits to prevent ambiguity in interpretation and harmonize the breeding goals with customer expectations.

We acknowledge the fact that breeding programs are dynamic and span multiple years, and so are related investments efforts. Also, depending on the breeding program and terms of the grants, investments can be used to facilitate diverse functions including germplasm development and seed dissemination to farmers. However, this study considered investments made in respective breeding pipelines in the year 2020 alone and as lumpsum without disentangling how they were appropriated to different functions in the breeding programs. Thus, readers are advised to interpret the results wit– caution - the investments data used may have not been exhaustive, and there is no assurance that they performed similar set of functions in the breeding programs. It is for these and other reasons that we also mask the absolute investment figures made in the respective pipelines in the given year.

## Conclusions

‘This paper sets out to answer the question: how well targeted are CIP-led potato and sweetpotato breeding efforts in several global commodity breeding pipelines toward delivering the greatest impact? The success of demand-driven breeding programs needs a strategic focus of the entire breeding and seed systems, and where informed integration of food security, nutrition and gender objectives into breeding programs is a significant part. We describe and analyze the market segments align them with corresponding breeding pipelines and recent investments. The analysis focus on poverty alleviation potentials, nutrition and gender-responsiveness of the breeding efforts in the respective breeding pipeline and market segments at high level. Breeding efforts in CIP were aligned to 23 market segments (14 on sweetpotato and 9 on potato) with varied needs and potential impacts. Overall, the results show that there is a significant scope for adjusting priorities and rebalancing investment with sensitivity to relative poverty and prevalence of malnutrition in target market segments. The analysis of nutritional indicators shows, for Africa, that there are higher rates of stunting, VAS coverage, and anemia prevalence in the markets segments where the WFSP is dominant. This finding underscores the need to enhance the promotion of more nutritious varieties such as biofortified OFSP in Africa. The very high prevalence of anemia and very low VAS coverage in Latin America similarly calls for biofortification of the potato in this segment. The nutrition profiles of all the categories of potato market segments also show that there is a greater opportunity to make significant nutritional impacts through the nutrition-sensitive interventions encompassing more investment in the table potato segment than in the processing segments since the former has a broader reach to stunted children and anemic women. We further conclude that breeding pipelines that do not yet include traits with gender implications, have an opportunity to challenge the low adoption ceiling by developing new varieties with these traits, particularly those associated with food quality. The stakeholders’ conclusion from the “*do no harm* analysis” that some desirable varietal traits, such as early maturity and higher yields, may exacerbate gender inequality driven by male-dominated commercialization confirms the value of a gendered customer and product profiling. Also, existing breeding strategies need intentional and context-specific gender-sensitive adjustments for greater impact. Future efforts to spearhead demand-driven breeding initiatives are recommended to undertake this type of multidimensional analysis to increase their awareness and relevance for poverty alleviation, nutritional impact, gender responsiveness, and inclusion of other relevant aspects as youth outreach and climate change resilience.

## Data availability statement

The raw data supporting the conclusions of this article will be made available by the authors, without undue reservation.

## Author contributions

SO, JO, JM and DO conceptualized the study, contributed to data gathering and analysis, and wrote the manuscript. HL-K, HC and VP sourced funds to facilitate the study, contributed to data gathering and edited the manuscript. PC, TM, MA, NS, WG, GM, RS, BY, SM, BO, DC, JA and GH contributed the primary content for one or more sections and provided additional editing support. All authors contributed to the article and approved the submitted version.

## Footnotes

1 We thank an anonymous reviewer for the suggestions used in justifying the focus on gender and nutrition in this paper.

2 FAOSTAT provides free access to food and agriculture data covering multiple commodities in different dimensions for 245 countries and territories and to the most recent years. http://www.fao.org/faostat/en/#data.

3 World Bank, Global Poverty Working Group. Data are compiled from official government sources or are computed by World Bank staff using national (i.e., country–specific) poverty lines. https://data.worldbank.org/indicator/SI.POV.DDAY https://data.apps.fao.org/catalog/dataset/world-development-indicators/resource/01e5892d-fe1d-45de-b126-25a0deb35f81?view_id=aad782ef-c891-424c-902a-70e59c633b3e.

4 A product of a combined effort by the FAO Statistics Division, the World Bank group and IFAD. It provides harmonized indicators and data on agricultural income and rural livelihoods disaggregated by sex, and other socio-economic statuses for the formulation of evidence-based development policies. Available here http://www.fao.org/in-action/rural-livelihoods-dataset-rulis/en/.

5 Head count ratio of people living on less than $1.9 a day corrected for 2011 purchasing power parity.

6 Malawi where there is high incidence of urban agriculture is one of the few exceptions.

7
http://www.fao.org/sustainable-developmentgoals/indicators/211/en/#:~:text=The%20prevalence%20of%20undernourishment%20(PoU,is%20expressed%20as%20a%20percentage.

8 SeeOrr et al. (2021) and Ashby and Polar (2021) for a detailed discussion of these tools.
